# Immunoregulation of microglial polarization: an unrecognized physiological function of α-synuclein

**DOI:** 10.1186/s12974-020-01940-z

**Published:** 2020-09-17

**Authors:** Na Li, Tessandra Stewart, Lifu Sheng, Min Shi, Eugene M. Cilento, Yufeng Wu, Jau-Syong Hong, Jing Zhang

**Affiliations:** 1 Department of Immunology, School of Basic Medical Sciences, Lanzhou University, Lanzhou, Gansu 730000 China; 2 Department of Pathology, School of Basic Medical Sciences, Peking University, Beijing, 100191 China; 3 Department of Pathology, University of Washington School of Medicine, Seattle, WA 98104 USA; 4 Neurobiology Laboratory, National Institute of Environmental Health Sciences, National Institutes of Health, Research Triangle Park, Durham, NC 27709 USA; 5 Department of Pathology, Zhejiang University First Affiliated Hospital and School of Medicine, Hangzhou, Zhejiang 310002 China

**Keywords:** Microglia, α-Synuclein, Neuroinflammation, Parkinson’s disease

## Abstract

**Background:**

Microglial function is vital for maintaining the health of the brain, and their activation is an essential component of neurodegeneration. There is significant research on factors that provoke “reactive” or “inflammatory” phenotypes in conditions of injury or disease. One such factor, exposure to the aggregated or oligomeric forms of α-synuclein, an abundant brain protein, plays an essential role in driving microglial activation; including chemotactic migration and production of inflammatory mediators in Lewy body (LB) diseases such as Parkinson’s disease. On the other hand, it is increasingly recognized that microglia also undergo changes, dependent on the cellular environment, that promote mainly reconstructive and anti-inflammatory functions, i.e., mostly desirable functions of microglia in a physiological state. What maintains microglia in this physiological state is essentially unknown.

**Methods:**

In this study, using in vitro and in vivo models, we challenged primary microglia or BV2 microglia with LPS + IFN-γ, IL-4 + IL-13, α-synuclein monomer, and α-synuclein oligomer, and examined microglia phenotype and the underlying mechanism by RT-PCR, Western blot, ELISA, IF, IHC, Co-IP.

**Results:**

We described a novel physiological function of α-synuclein, in which it modulates microglia toward an anti-inflammatory phenotype by interaction with extracellular signal-regulated kinase (ERK) and recruitment of the ERK, nuclear factor kappa B (NF-κB), and peroxisome proliferator-activated receptor γ (PPARγ) pathways.

**Conclusions:**

These findings suggest a previously unrecognized function of monomeric α-synuclein that likely gives new insights into the pathogenesis and potential therapies for Lewy body-related diseases and beyond, given the abundance and multiple functions of α-synuclein in brain tissue.

## Background

Accumulating evidence supports a role of microglial dysfunction in a wide range of neurological disorders, including in the pathogenesis and progression of neurodegenerative diseases such as Lewy body (LB) disorders like Parkinson’s disease [1]. In their quiescent state, microglia are ramified cells with multiple branches and processes, which dynamically survey the brain’s microenvironment, responding to signals by performing phagocytic scavenging, immune surveillance, and maintenance of normal brain functioning and tissue integrity [2–4]. Once an insult occurs, microglia can quickly switch from their quiescent state to an activation state in order to maintain brain homeostasis [5]. Activated microglia may develop into states that can be broadly categorized as pro-inflammatory or anti-inflammatory conventionally conceptualized as “M1” or “M2” phenotypes, respectively [6, 7]. More recently, it becomes increasingly clear that such a dichotomy is oversimplified, with evidence supporting multiple subpopulations of polarized microglia exerting unique physiological and biological features. Yet, the broad M1 and M2 classification remains a useful concept in differentiating functional state of microglia [8]. The former phenotype produces inducible nitric oxide synthase (iNOS) and secretes pro-inflammatory cytokines such as interleukin 1 β (IL-1β), tumor necrosis factor α (TNF-α), and interleukin 18 (IL-18), which elicit an immune response, and potentially result in damage to neurons through exposure to these secreted neurotoxic substances. In contrast, the anti-inflammatory phenotype is typically associated with dampening of inflammation, repair of damaged tissues, and resolution of injuries. In this state, microglia express arginase 1 (ARG-1), with downstream products of polyamines [9] associated with tissue repair, and secrete anti-inflammatory cytokines, such as IL-10, IL-4, or IL-13. How microglia are maintained at physiological state remains to be characterized, but abating the excess microglia-mediated inflammation and regulating microglia toward largely an anti-inflammatory phenotype may be a promising therapy for controlling neuroinflammation and a potential treatment of neurodegenerative diseases [10–14].

α-Synuclein (α-Syn), a soluble protein prone to formation of oligomers and aggregates, is implicated in LB diseases. It is prominently expressed in regions of adult CNS including the cerebral cortex, midbrain, amygdala, and olfactory bulb [13, 14]. It is highly enriched at presynaptic terminals, and thought to be engaged in binding lipids and regulating the release of synaptic vesicles, especially in dopamine neurotransmission [10]. Because its aggregated form is a primary component of the LBs that define the brain pathology of Parkinson’s disease and related disorders [15], and extensive data suggests functional roles of α-Syn oligomers/aggregates in Parkinson’s disease etiology, the participation of its pathological (oligomeric/aggregated) forms in disease is better understood than the physiological roles of the monomer. Further, aggregated α-Syn is a potent activator of microglia, provoking transition to a pro-inflammatory phenotype [16, 17]. Intriguingly, data demonstrating that the neurotoxicity of aggregated α-Syn in vitro was dependent on the presence of microglia suggests that its deleterious effects on neurons may be largely mediated through microglial inflammatory processes [16, 18]. Despite this well-known microglial effect of aggregated α-Syn, the effects of non-pathological forms of α-Syn, which are much more abundant compared to aggregated species, on microglia have not been investigated in detail. Therefore, in this study, we examined the potential role of monomeric α-Syn in mediating microglia polarization to develop further insights into the connection between α-Syn, neuroinflammation, and Parkinson’s disease pathogenesis.

## Methods

### Animals

SNCA-KO mice (B6;129X1-Snca^tm1Rosl^/J), wild-type mice (ICR/HaJ), and dbl-PAC-Tg(SNCAA53T);SNCA^−/−^ mice were purchased from the Jackson Laboratory. SNCA-KO and dbl-PAC-Tg(SNCAA53T);SNCA^−/−^ mice lack endogenous α-Syn expression in brain, while the latter has peripheral expression of the human A53T mutant form [14, 19]. The mice were maintained in a specific pathogen-free facility.

Lipopolysaccharide (LPS; Sigma, L2630; ≥ 500,000 Endotoxin Units/mg) was reconstituted in water to 1 mg/ml, then diluted with phosphate-buffered saline (PBS; GIBCO, 10010023) to a dose of 3 mg/kg in a volume of 200 μl. LPS was administered via intraperitoneal (i.p.) injections when mice (SNCA-KO) were 8 weeks old at *t* = 0 h, *t* = 6 h, and *t* = 24 h. For α-Syn monomer injections, a dose of 5 mg/kg with a volume of 200 μl was given via tail vein intravenous injection (IV) following the last LPS injection and mice were sacrificed at *t* = 36 h. Proteins of mice brain were extracted by animal tissue protein extraction kit (Sangon Biotech, C500006).

For the Parkinson’s disease model, 15 4-month-old dbl-PAC-Tg(SNCAA53T);SNCA^−/−^ mice were randomly divided into three groups. This strain models early human Parkinson’s disease, showing a gastrointestinal dysfunction without major central nervous system pathology [19, 20]. Mice received intraperitoneal injection of 1-methyl-4-phenyl-1,2,3,6-tetrahydropyridine (MPTP; Selleck Chemicals LLC, S4732) at 30 mg/kg/day (once daily for 5 successive days) [21–23], with or without IV injection of α-Syn monomers at a dose of 5 mg/kg following each MPTP injection. Mice were sacrificed 12 h after the last injection. An equivalent volume of saline injection served as a control [24]. For each mouse, the brain was cut in half along the sagittal plane. One part was fixed in 4% paraformaldehyde (PFA) for 24 h and dehydrated in 30% sucrose for 48 h before frozen section preparation, while the other part was used for protein extraction in substantia nigra (SN), striatum (ST), or cerebral cortex by animal tissue protein extraction kit (Sangon Biotech, C500006).

### α-Syn monomer and aggregates preparation

α-Syn protein (HNAE, 12093) was diluted and identified according to the manufacturer’s instruction.

α-Syn aggregates were prepared according to a previously published protocol [16, 25]. Briefly, purified α-Syn was resuspended in water at a concentration of 1 mg/ml, incubated at 37 °C with agitation for 7 days to generate α-Syn aggregates. Isoforms were characterized by Western blot. Monomer preparations showed only a single band around 15 kDa (the expected molecular weight of monomeric α-Syn), while the oligomeric aggregates showed multiple bands ranging from 35 to 135 kDa on the gel (Fig. s1a), consistent with previous studies [16, 26].

### Primary microglia isolation

Primary microglia were generated from neonatal ICR mice and cultured as published [16, 27]. Primary microglia culture purity was assessed by staining for both the microglia marker iba-1 and the astrocyte marker glial fibrillary acidic protein (GFAP; for possible astroglial cell contamination) (Fig. s1b). The purity of microglia cultures was determined to be > 98% (Fig. s1c).

### Microglia culture and treatment

Microglial cell line BV2, which was generated by infecting mouse primary microglia culture with retrovirus J2 carrying a v-raf/v-myc oncogene [28], were used in some experiments. BV2 secreted IL-1 and TNF-α following appropriate stimulation and retained most of morphological, phenotypical, and functional properties as that of primary microglia. To confirm results, BV2 cells were used for some experiments before validation in primary cells or animals. Cell line or primary microglia were plated onto 6-well plate (Corning, 3516) at a density of 5 × 10^5^ per well with F12/DMEM containing 10% fetal bovine serum (FBS) free of Penicillin-Streptomycin (PS); the cells were incubated at 5% CO_2_, 37 °C overnight, and then the media was replaced with F12/DMEM free of FBS and PS for further stimulation. All FBS was heat inactivated at 56 °C for 30 min before use in culture media.

Stimulating factors included LPS (100 ng/ml) plus interferon γ (IFN-γ) (Peprotech, 315-05) (10 ng/ml), IL-4 (Peprotech, 214-14) (20 ng/ml) plus IL-13 (Peprotech, 210-13) (10 ng/ml), or physiological concentration of α-Syn (50 nM, 100 nM, 250 nM) for 6 h, 12 h, or 24 h [16, 27]. The supernatants of media were collected and filtrated through 0.45 μm filter. Nitrate concentration was measured using Nitric Oxide Assay Kit (Beyotime, S0021). For mechanism related molecules detection, cells were stimulated for 12 h only.

For treatment of primary microglia with α-Syn oligomer, the procedure was the same and stimulation was for 12 h. Pre-treatment of microglia by α-Syn monomer was conducted 2 h before oligomer stimulation. Longer pre-treatment (6 h, 12 h) by α-Syn monomer (100 nM) was conducted to further investigate the monomeric α-Syn in attenuating pro-inflammatory effect of oligomeric α-Syn, before oligomeric α-Syn was added, monomeric α-Syn in culture system was washed out with PBS three times, and incubation of oligomeric α-Syn lasted for either another 6 h or 12 h.

For extracellular signal-regulated kinase (ERK) phosphorylation induced by honokiol (sigma, H4914), primary microglia were treated with 10 μM and 20 μM honokiol for 90 min, either after or before treatment with α-Syn (100 nM) for 30 min.

### Real-time PCR

Total RNA was isolated from microglia using TRIZOL reagent (Invitrogen, 15596-026) according to the manufacturer’s instruction. First-strand cDNA was synthesized from about 2 μg total RNA using FastQuant RT Kit with gDNase (TIANGEN, KR106). Then, 1 μl reverse-transcribed cDNA was used in real-time PCR with Hieff™ qPCR SYBR® Green Master Mix (Low Rox) (YENSEA, 11203ES08). Expressions of GAPDH, Ubc, and 18s rRNA (the mean of the three housekeeping genes) were used as a control for normalizing the amounts of cDNA. The primers used were as follows: iNOS, forward primer: ACCTTGTTCAGCTACGCCTT, reverse primer: CATTCCCAAATGTGCTTGTC; ARG-1, forward primer: CAACTCTTGGGAAGACAGCA, reverse primer: GTCAGTCCCTGGCTTATGGT; CD206, forward primer: TGATTGTTGATTGCCCACTT, reverse primer: AATCTGCAGGGTTGACATGA; CD16/32, forward primer: GGCTCATTGGACACAACAAC, reverse primer: TCCTATCAGCAGGCAGAATG; GAPDH, forward primer: AATGTGTCCGTCGTGGATCT, reverse primer: AGACAACCTGGTCCTCAGTG; Ubc, forward primer: GGTCGATGCCAGTGAAACTAGCAAGAAGG, reverse primer: CCCCCAGCACACCCTTGAACAAGCACAAG; 18s rRNA, forward primer: GGCGGTACTATTTTGTTGGT, reverse primer: AGTCGCCATCGTCAATGGTCA. All results were analyzed using 2^-△△Ct^ and were presented as mean + s.e.m.

### Enzyme-linked immunosorbent assay

The supernatant of microglia stimulated with LPS + IFNγ, IL-4 + IL-13, α-Syn, or control were collected and filtered with 0.45 μm filter, IL-10, TNF-α, and IL-1β were examined by enzyme-linked immunosorbent assay (ELISA) kit (Mouse-IL-10-ELISA-Kit, KE10008, Mouse-TNF-alpha-ELISA-Kit, KE10002, Mouse-IL-1-beta-ELISA-Kit,KE10003) according to the manufacturer’s instructions.

### Fluorescence staining

Cells were plated overnight on microscopy grade petri dishes (Jet biofilm, BDD002035) at a density of 2 × 10^5^/ml with F12/DMEM containing 10% FBS, and then media were replaced by F12/DMEM free of FBS and stimulated with LPS + IFN-γ or IL-4 + IL-13 or α-Syn for 12h. Cells were stained with the first antibodies including Iba-1 (1:250, Wako, 019-19741), GFAP (1:250, Abcam, ab68428), IL-1β (1:250, Santa Cruz, sc-7884), ARG-1 (1:250, Santa Cruz, sc-271430), iNOS (1:250, Abcam, ab-178945), and NF-κB p65 (1:250, CST, 8242). On the next day, cells were stained with fluorescein-labeled antibodies including Alexa Fluor 594-conjugated goat ant-rabbit IgG (H + L) (1:500, Thermo Fisher, A-11037), Alexa Fluor 488-conjugated goat ant-mouse IgG (H + L) (1:500, Abcam, ab150117), Alexa Fluor 488-conjugated goat ant-rabbit IgG (H + L) (1:500, Abcam, ab150077), and DAPI. Images were acquired under confocal microscope (ZEISS, LSM710).

For frozen sections, the protocol used was similar to that of cell staining with the minimal difference that before nuclear staining, the frozen sections were immersed in 0.3% Sudan Black dissolved in 70% EtOH for 45 min to reduce auto-fluorescence. iNOS (1:250, Abcam, ab49999) and IL-1β (1:250, Santa Cruz, sc-52012) were also used at various points during parallel preparations.

For Parkinson’s disease model mice (dbl-PAC-Tg(SNCAA53T);SNCA^−/−^), cardiac perfusion was performed with 20 ml chilled PBS, then the brain was cut in half at the sagittal plane, with one part used for protein extraction and the other for frozen section preparation. Coronal sections of 30 μm starting at 2.46 mm from Bregma and ending at 4.04 mm from Bregma were collected serially and separated one section from the consecutive 120μm for IHC or IF [29].

### Immunohistochemistry and cytotoxicity assay

The supernatants of microglia stimulated with LPS + IFN-γ, α-Syn, oligomer, pre-α-Syn + oligomer, or control were collected and filtered through 0.45 μm filter (Millipore, R1EA), and stored at − 80 °C until use. SH-SY5Y neurons were plated on coverslips at a density of 2 × 10^5^/ml with DMEM/F12 containing 10% FBS overnight, and then the media were changed with conditioned media from LPS + IFN-γ, α-Syn, oligomer, pre-α-Syn + oligomer (5 pg/ml and 400 pg/ml), or control stimulated microglia and co-cultured for 24 h. The procedure for staining dopaminergic neurons in SN with tyrosine hydroxylase (TH) (1:250, Millipore, AB152) and the HRP-labeled antibody (HRP-conjugated goat anti-rabbit IgG(H + L), 1:1000, Abcam, ab6721) and quantification of dopaminergic neurons were performed as previously published [16, 29]. F-actin of SH-SY5Y was stained with TRITC phalloidin (YEASEN, 40734ES75) and images were acquired using × 20 magnification. To quantify neurite length, neurites were selected at the origin site at the neuronal cell body, and then following along the longest neurite of each cell with the scale tool in Adobe Photoshop followed. Neurite lengths were normalized to control cells using segmented line in ImageJ (1.52a) software. Cytotoxicity was assessed using a CCK-8 kit according to the manufacturer’s instructions; specifically, cell viability (%) = [OD(treatment)-OD(blank)] / [OD(control)- OD(blank)] × 100.

### Western blotting

Proteins were extracted from microglia using cell lysis buffer (RIPA, HARVEYBIO, C1503; Halt Protease & Phosphatase Inhibitor Cocktail (× 100), Thermo, 78444), and a BCA Protein Assay Kit (Applygen, P1511) according to the manufacturer’s instruction determined the concentration. Western blot was carried out as previously described. Antibodies used included iNOS (1:1000, Abcam, ab-178945), ARG-1 (1:1000, Santa Cruz, sc-271430), ERK1/2 (1:1000, Abcam, ab184699), p-ERK1/2 (1:1000, Abcam, ab76299), NF-κB (1:1000, CST, 8242), p-NF-κB (1:1000, CST, 3033), IKKα/β (1:1000, Abcam, ab178870), IKBα (1:1000, Abcam, ab32518), p-IKBα (1:1000, Abcam, ab133462), PPARγ (1:1000, Santa Cruz, sc-81152), GAPDH (1:1000, Trans, HC301), and Histone H3 (1:1000, Abcam, ab201456). The secondary antibodies were HRP-conjugated goat anti-rabbit IgG (H + L) (1:5000, Biodragon, BF03008) and HRP-conjugated goat anti-mouse IgG (H + L) (1:5000, Biodragon, BF03001).

For nuclear and cytoplasmic protein detection, ProteinExt Mammalian Nuclear and Cytoplasmic Protein Extraction Kit (Trans, DE201) was used according to the manufacturer’s instructions, and then Western blot was carried out as above.

For α-Syn monomer and oligomer identification, 25 μg α-Syn monomer or oligomer was loaded onto an SDS-PAGE. Anti-alpha-synuclein (1:1000, Abcam, ab138501) and HRP-conjugated goat anti-rabbit IgG (H+L) (1:5000, Biodragon, BF03008) were used to recognize the target band.

The densitometry of Western blot was calculated by Adobe Photoshop CC. GAPDH served as normalization control for total target proteins, and Histone H3 served as normalization control for nuclear proteins.

### Co-IP

Microglia were plated on 10-cm dish with F12/DMEM containing 10% FBS at a 70–80% confluent overnight, then media were changed to F12/DMEM free of FBS and stimulated with 250 nM α-Syn for 15 min, 30 min, 1 h, and 2 h. Cell protein was extracted by 1% NP-40 (Beyotime, ST366) in PBS containing 1% PIC (Pierce, 87786). Cells were lysed and collected into a 1.5 ml tube and centrifuged at 14,000 rpm at 4 °C for 5 min. The supernatants of whole cell lysates were regarded as pre-IP or input. Further, 10 μg mouse anti-human α-Syn antibody (Santa Cruz sc-12767) or mouse control IgG (Abcam, ab18447) was incubated overnight with 1000 μg protein at 4 °C overnight. Then, 100 μl Protein A + G Agarose (Beyotime, P2012) was washed twice with 200 μl 1% NP-40 in PBS, centrifuged at 14,000 rpm, 4 °C for 2min. The beads were added to protein and antibody reaction system and rocked at 4 °C for 6 h, collected by centrifugation at 14,000 rpm, 4 °C for 2 min, and then washed twice with 1% NP-40 in PBS. The protein-antibody complex was then loaded onto SDS-PAGE. Rabbit ERK (1:1000, Abcam, ab184699) and rabbit anti-human α-Syn (1:1000, Abcam, ab138501) were used to detect the specific protein band on PVDF membrane.

### Statistical analysis

ANOVA analysis or t test was performed for comparisons among groups using GraphPad Prism 5. Data were shown as their mean ± s.e.m. *p < 0.05 was considered a statistically significant difference.

## Results

### Monomeric α-Syn induces microglia toward an anti-inflammatory phenotype

We first examined the effects of α-Syn monomer within a range of concentrations on the phenotype of BV2 microglial line cells. As positive controls, BV2 cells were induced toward either a pro-inflammatory phenotype, using lipopolysaccharide (LPS) + IFN-γ, or an anti-inflammatory phenotype, using IL-4 + IL-13 [30]. Successful induction was demonstrated by examination of phenotype markers: iNOS, which under M1 polarization converts arginine into citrulline to produce nitric oxide (NO), and ARG-1, which under M2 polarization converts arginine into ornithine and urea [31, 32]. Monomeric state of α-syn before and after the incubation period was confirmed (Fig. s1).

**Fig. 1:**
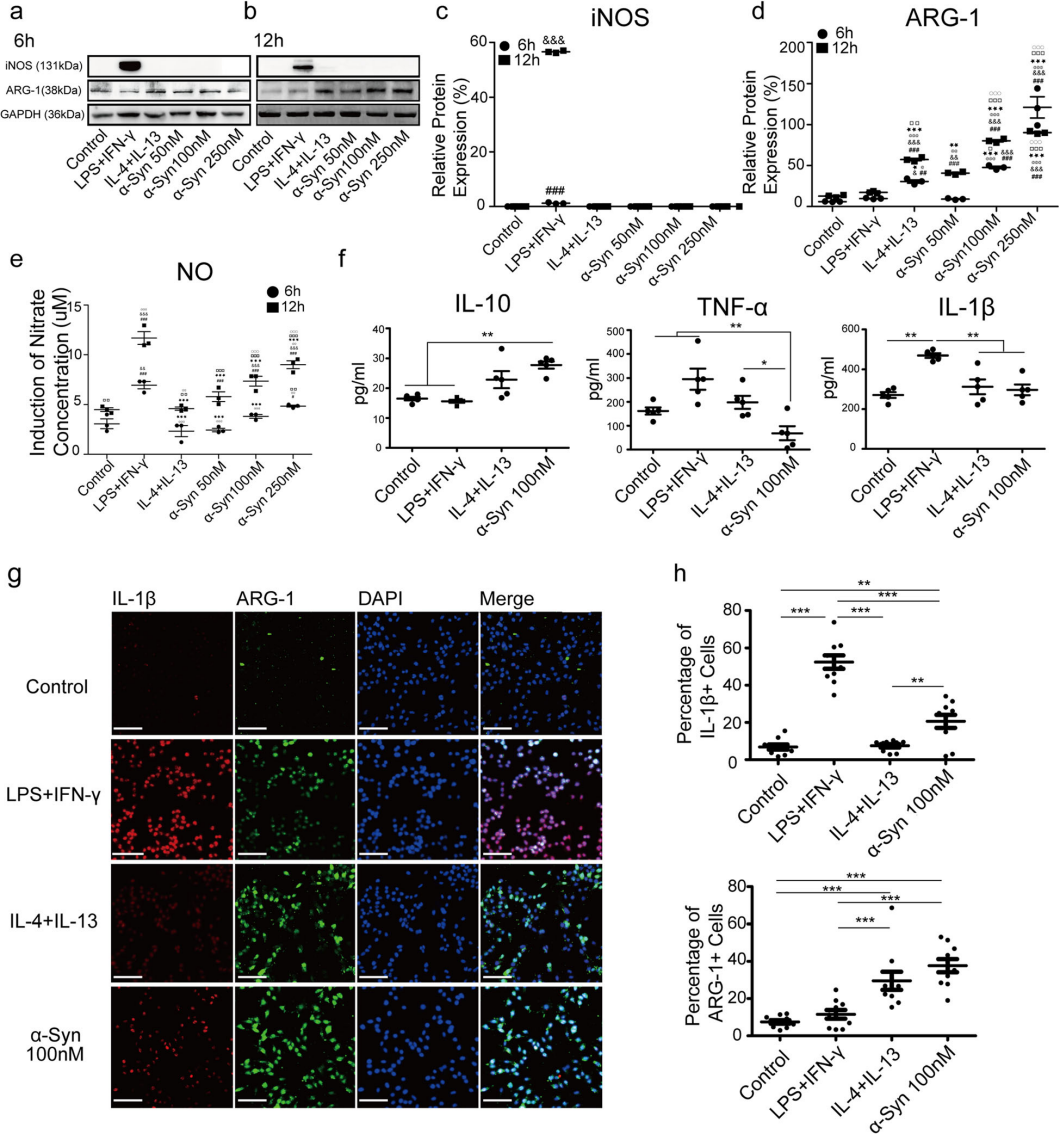
Monomeric α-Syn induces microglia towards anti-inflammatory phenotype. **a**, **b** α-Syn promotes ARG-1 expression in a dose-dependent manner without affecting iNOS in primary microglia following 6h or 12h treatment. **c**, **d** Quantitative analyses of iNOS and ARG-1 in immunoblots (*n* = 3 independent experiments). **e** Nitrate concentration in supernatant at 6 h and 12 h under different concentrations of α-Syn treatment in primary microglia (*n* = 3 with 5 replications. 6 h : asterisk vs. control, at sign vs. LPS + IFN-γ, white square vs. IL-4 + IL-13. 12 h: ampersand vs. control, black star vs. LPS + IFN-γ, whote circle vs. IL-4 + IL-13.). **f** Levels of cytokines IL-10, TNF-α, and IL-1β in supernatant of BV2 by ELISA (*n* = 3 with 5 replications). **g** Co-localization of IL-1β (red) and ARG-1 (green) in BV2 microglia with treatment of α-Syn (100 nM) for 12 h (scale bar = 10 μm). **h** Quantification of the percentage of IL-1β and ARG-1 positive cells (*n* = 5 replications each containing 15-20 fields). One-way ANOVA with Newman Keuls multiple comparison test. Bar graphs show mean + s.e.m. *p < 0.05, **p < 0.01***, *p* < 0.001

We further confirmed the expression of microglial polarization markers at the protein level in primary microglia. Treatment with monomeric α-Syn for 6 h or 12 h resulted in an increase in ARG-1 expression, but no detectable iNOS expression, collectively indicative of an anti-inflammatory state (Fig. 1a-d). Nitrate concentrations measured in culture media were largely in accordance with the iNOS levels in primary microglia, i.e., monomeric α-Syn, particularly at lower concentrations, did not increase the nitrate production (Fig. 1e), in contrast to LPS + IFN-γ, further suggesting that the α-Syn monomer treatment at low levels and shorter times did not induce microglia into an inflammatory phenotype. However, when high concentrations were used at longer time points (Fig. s2), nitrate levels were elevated compared to control and IL-4 + IL-13 treatment, possibly due to the formation of α-Syn oligomers under these conditions, and suggesting that the dose response of the α-Syn effect is dependent on the balance between monomer and oligomer formation. Similar results were observed when the effect of α-syn monomer on expression of inflammation-related genes was measured using mRNA in the BV2 microglial cell line (Fig. s2).

When examining cytokines, the pro-inflammatory cytokine TNF-α was dramatically reduced (*p* < 0.05 compared with control or LPS + IFN-γ treatment), while IL-1β remained similar to the levels in control and IL-4 + IL-13 treatment. In contrast, the anti-inflammatory cytokine IL-10 was elevated by treatment with α-Syn monomer (*p* < 0.05 compared with control or LPS + IFN-γ treatment) (Fig. 1f). This suggested that monomeric α-Syn regulated microglia toward an anti-inflammatory phenotype when cells were treated for 12 h.

These results were further supported by immunofluorescence experiments in the BV2 microglial cell line. Incubation with α-Syn monomers at 100 nM induced ARG-1, but not IL-1β (another marker of microglial inflammation) (Fig. 1g), within BV2 cells at 12 h. The percentage of IL-1β^+^ cells in the control-, IL-4 + IL-13-, and monomeric α-Syn-treated groups were 6.80%, 7.45%, and 20.63%, respectively. All were lower than that in the LPS + IFN-γ group (52.44%). The percentage of ARG-1^+^ cells in monomeric α-Syn-treated microglia was 37.70%, far higher than that in control and LPS + IFN-γ groups (7.48%, 11.57%, respectively) (Fig. 1h), and similar to the IL-4 + IL-13 group (62.63%). These results further suggest that treatment with α-Syn monomer shifts the expression pattern of the microglial population toward the anti-inflammatory state.

### Monomeric α-Syn decreases induction of microglial pro-inflammatory phenotype and neurotoxicity by oligomeric α-Syn

**Fig. 2:**
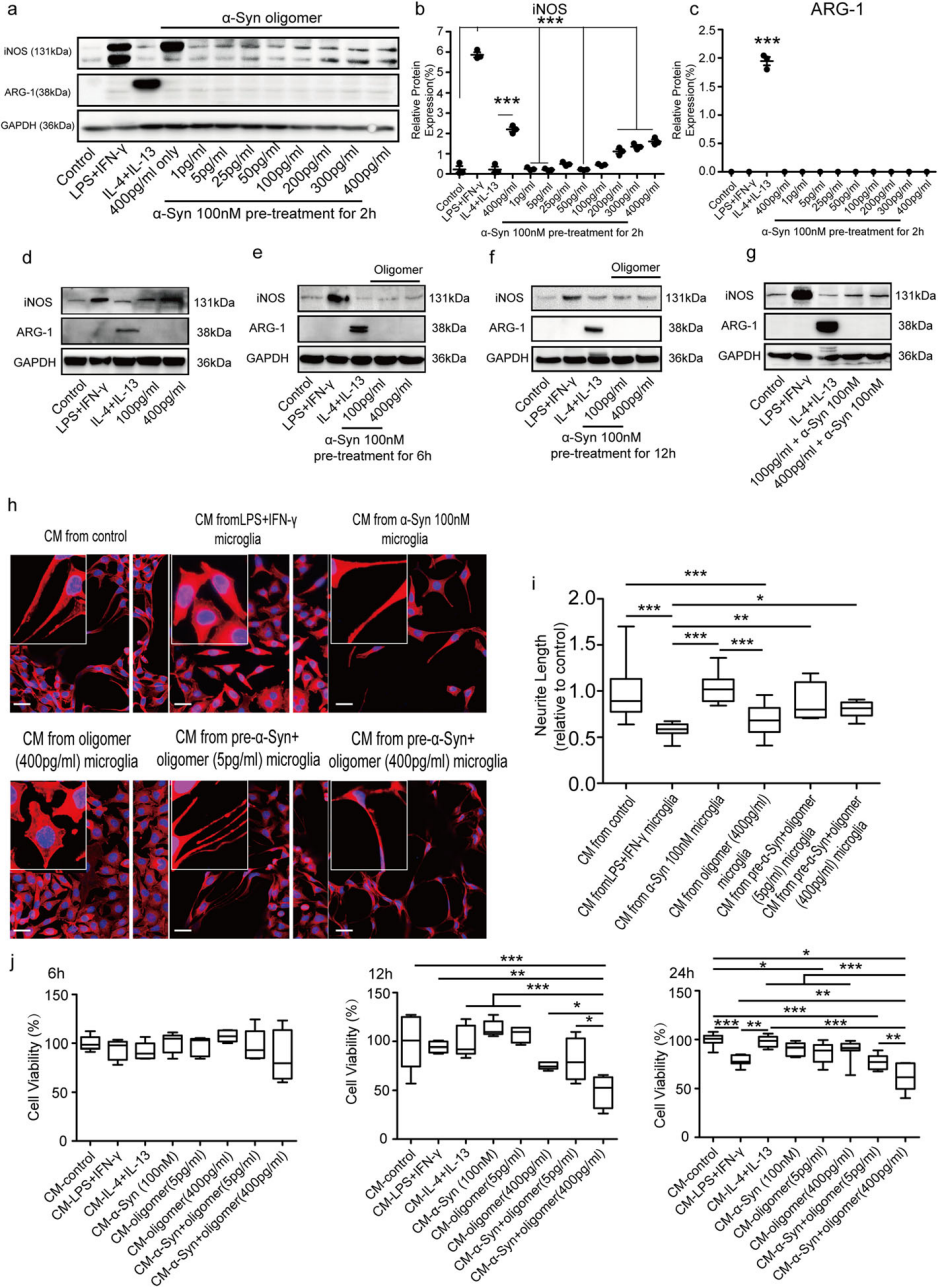
Treatment with monomeric α-Syn attenuates pro-inflammatory and neurotoxic effects of oligomers. **a** α-Syn (100 nM) pre-treatment (2 h) reduces iNOS expression in microglia following oligomeric α-Syn treatment (*n* = 3 independent experiments). **b** Densitometric analysis of relative intensity iNOS expression. **c** Densitometric analysis of relative intensity ARG-1 expression. **d** Oligomeric α-Syn (100 pg/ml and 400 pg/ml) promoted iNOS expression in microglia incrementally. **e** α-Syn (100 nM) pre-treatment (6 h) reduces iNOS expression in microglia following oligomeric α-Syn treatment for another 6 h. **f** α-Syn (100 nM) pre-treatment (12 h) reduces iNOS expression in microglia following oligomeric α-Syn treatment for another 12 h. **g** Co-treatment of microglia by monomeric and oligomeric α-Syn for 12 h induces iNOS expression. **h** F-actin of neurons was stained with TRITC phalloidin under treatment by conditioned media. (Scale bar = 10 μm). **i** Neurite lengths are estimated under different conditioned media treatment (*n* = 3 replications each containing 15–20 fields). **j** Cell viabilities of neurons are detected by CCK-8 assay after treatment with monomeric α-Syn, α-Syn oligomer, or pre-treatment α-Syn plus oligomer conditioned media from 6 h, 12 h, and 24 h (*n* = 5 independent experiments with 5 replications). One-way ANOVA with Newman Keuls multiple comparison test. Bar graphs show mean + s.e.m.*p < 0.05; **p < 0.01***; *p* < 0.001

In previous studies [16, 33], treatment of microglia with α-Syn oligomers provoked a pro-inflammatory response. We therefore sought to determine whether exposure to monomeric α-Syn at 100 nM might alter the balance of microglial activation away from the pro-inflammatory effects of oligomeric α-Syn. We pretreated microglia with monomeric α-Syn for 2 h, before stimulation with differing concentrations of oligomers. As in previous studies [16, 25], α-Syn oligomers promoted a pro-inflammatory microglial phenotype, with treatment of microglia with α-Syn oligomers leading to a dose-dependent induction of iNOS protein expression (Fig. s3 a-b). Similarly, higher concentrations of oligomers effectively promoted iNOS mRNA expression in BV2 cells, and ARG-1 mRNA remained low under oligomer treatment compared with IL-4 + IL-13 treatment (Fig. s3c). The percentage of iNOS^+^ cells in oligomer-treated BV2 cells (39.14%) was higher than that of control (3.15%) and IL-4 + IL-13 treated BV2 cells (11.80%), while the percentage of ARG-1^+^ cells in oligomer treated microglia (20.74%) was lower than that of IL-4 + IL-13 treated cells (30.55%) (Fig. s3d-e). Intriguingly, pre-treatment (2 h) of microglia with α-Syn attenuated oligomer-induced iNOS expression (Fig. 2a-c). Longer pre-treatments (6 h and 12 h) strongly reduced iNOS expression in microglia even when monomeric α-Syn was eliminated by washing out before oligomeric α-Syn was added (Fig. 2d-f). Simultaneous co-stimulation of microglia by oligomeric and monomeric α-Syn for 12 h resulted in elevated iNOS expression in microglia (Fig. 2g). These results suggest that pre-treatment with α-Syn monomer may ameliorate pro-inflammatory effects induced by α-Syn oligomer to some extent.

A previous study demonstrated that neither monomeric nor oligomeric α-Syn mediated direct toxicity on SH-SY5Y neurons, but rather neurotoxicity was observed when microglia were activated by aggregated α-Syn [27]. Thus, we also examined the indirect effects of BV2 cells treated with monomeric α-Syn on SH-SY5Y cells. To accomplish this, we collected conditioned media from BV2 cells treated with monomeric α-Syn, oligomeric α-Syn, LPS + IFN-γ or pre-treatment with monomeric α-Syn followed by oligomer, and exposed cultured SH-SY5Y neuronal cells to it for 24 h. Media from monomeric α-Syn-treated microglia maintained neurites, which showed no statistical difference compared with that of control (Fig. 2h, i). However, SH-SY5Y cells exposed to media from BV2 cells treated with oligomers (400 pg/ml) only or monomeric α-Syn pre-treatment plus low/high concentration of oligomer (5 pg/ml or 400 pg/ml) treatment had significantly shorter neurites (Fig. 2h, i) and reduced viability (Fig. 2j). Unlike oligomer, monomeric α-Syn does not induce microglia-like cells toward a neurotoxic effect on SH-SY5Y cells, but the relationship of effects when both monomer and oligomer are present are less clear.

### Monomeric α-Syn may regulate microglia toward anti-inflammatory phenotype through ERK, NF-κB, and PPARγ

**Fig. 3:**
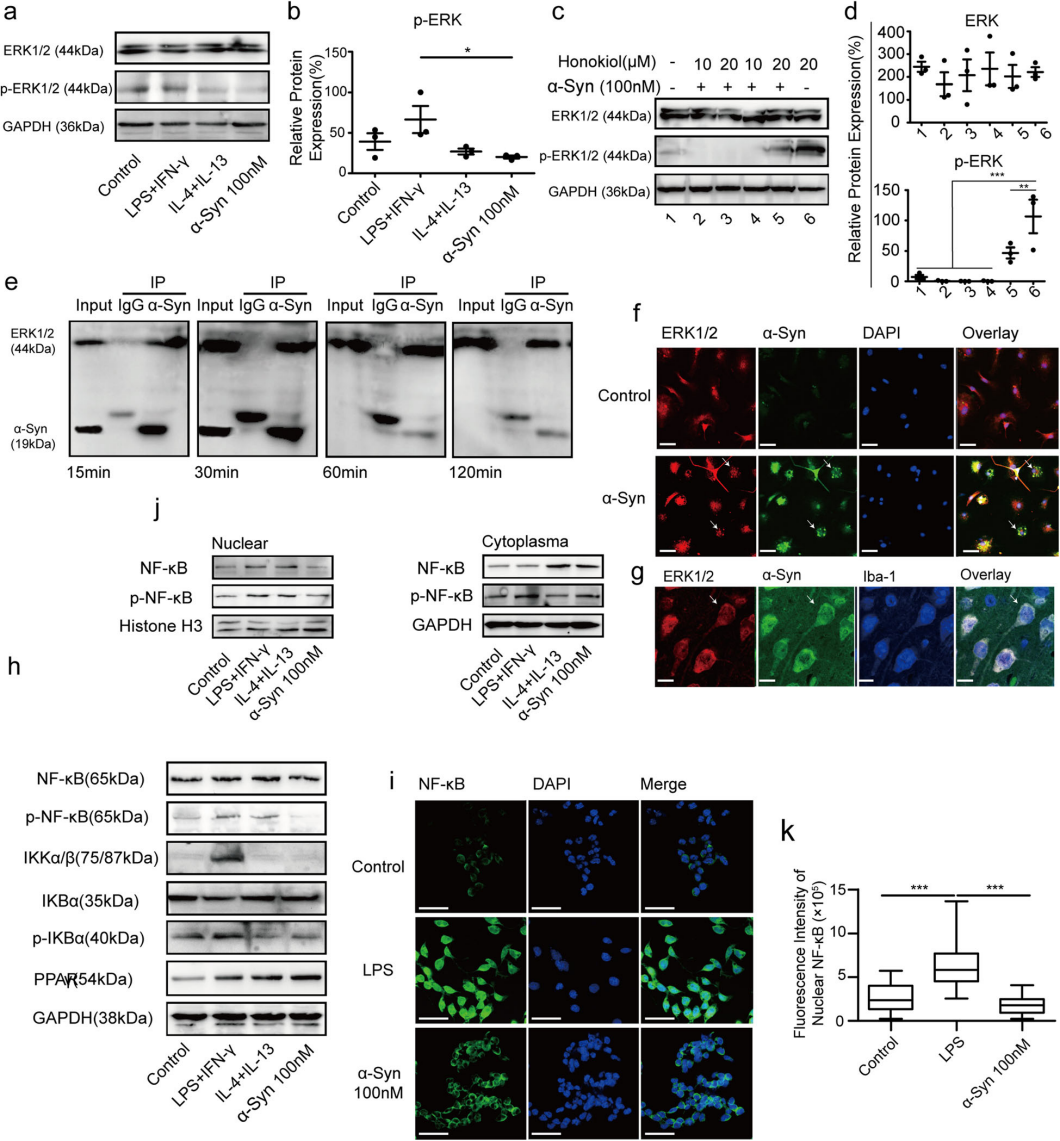
Monomeric α-Syn regulates microglia towards anti-inflammatory phenotype through ERK, NF-κB, and PPARγ pathways. **a** α-Syn (100 nM) treatment of primary microglia inhibits p-ERK without obvious effect on total ERK. **b** The densitometric analysis shows significant decrease of p-ERK under α-Syn treatment. **c** Inhibition of honokiol inducing ERK phosphorylation is observed under monomeric α-Syn treatment (*n* = 3). **d** Quantification of ERK and p-ERK. (Band 1: microglia without stimulation. Band 2, 3: pre-treatment with α-Syn for 30min, then treated with 10 μM and 20 μM honokiol for 90 min, respectively. Band 4, 5: post-treatment with α-Syn for 30min following 10 μM and 20 μM honokiol treatment for 90 min. Band 6: microglia treated with 20 μM honokiol only for 90 min). **e** Co-IP of BV2 cell-generated protein is performed using α-Syn and ERK antibodies, and the interaction of α-Syn with ERK is detected at 15 min, 30 min, 1 h, and 2 h post α-Syn treatment (*n* = 3). **f** Co-localization of α-Syn (green) and ERK (red) in primary microglia after treatment with α-Syn for 15 min (Scale bar = 100 μm, *n* = 3 replications). **g** Co-localization of α-Syn (green) and ERK (red) in microglia of mouse brain (scale bar = 10μm, *n* = 5 mice). **h** α-Syn (100 nM) treatment prevents NF-κB expression and induces PPARγ. NF-κB activation related proteins IKKα/β and p-IKBα also show a significant decrease in primary microglia after treatment by α-Syn (*n* = 3 independent experiments). **i** NF-κB (green) is mainly in the cytoplasm of primary microglia with α-Syn treatment compared with that of LPS treatment, which shows a condensed expression in the nucleus confirmed by confocal microscopy (Scale bar = 10 μm. *n* = 5). **j** α-Syn (100nM) treatment inhibits NF-κB translocation into the nucleus of primary microglia with reduction of NF-κB and p-NF-κB in the nucleus (*n* = 3 independent experiments). **k** Fluorescence intensity of NF-κB in nuclear in BV2. One-way ANOVA with Newman Keuls multiple comparison test. Bar graphs show mean + s.e.m.*p < 0.05; **p < 0.01; ***p < 0.001

Activation of ERK by phosphorylation is a key step in regulation of microglial pro-inflammatory phenotype [34, 35], and is known to play a role in microglial pro-inflammatory processes in a Parkinson’s disease mouse model [36, 37]. We hypothesized that this pathway might be involved in promotion of the anti-inflammatory phenotype by monomeric α-Syn. To accomplish this, we measured the levels of ERK and p-ERK1/2 in cultured primary microglia exposed to it. While neither monomeric nor oligomeric α-Syn altered the levels of total ERK (Figs. 3a and s4). The significant reduction in p-ERK levels (Fig. 3a, b) suggested that ERK signaling might indeed be regulated by monomeric α-Syn. Similar experiments were performed to determine whether other pathways might also be altered by monomeric α-Syn, but no other pathways appeared to be noticeably altered (Figs. s5 and s6). We then applied honokiol, an agonist enhancing the phosphorylation of ERK. After a 30-min pre-treatment with monomeric α-Syn, the increase in p-ERK induced by honokiol was significantly attenuated in the pre-treatment condition (Fig. 3c, d).

Having determined that ERK signaling is likely involved, we next investigated the mechanism by which α-Syn interacts with ERK. Previous results from quantitative proteomics indicate that ERK is one of the multitudinous proteins associated with soluble α-Syn [38, 39], prompting us to speculate that α-Syn may directly interact with ERK protein. In this study, we first treated BV2 cells with α-Syn at different time points (15 min, 30 min, 60 min, and 120 min), then performed co-IP to test whether α-Syn monomers could capture ERK originating from them. Interaction of α-Syn and ERK was observed at all stimulation time points examined (Fig. 3e). Similarly, immunofluorescent staining also showed co-localization of α-Syn and ERK around the nucleus in primary microglia (Fig. 3f), in Iba-1 positive cells of mouse brain (Figs. 3g and s4b), consistent with a direct or indirect interaction with ERK by both endogenous and exogenous α-Syn monomer.

We also probed how monomeric α-Syn affects activation of the transcription factor NF-κB, which induces pro-inflammatory phenotype in microglia [30, 40]. Although monomeric α-Syn had no notable effect on the levels of total NF-κB in primary microglia, the levels of p-NF-κB (activated NF-κB) were reduced (Figs. 3h and s6c). Similarly, separation of nuclear and cytoplasmic protein showed both NF-κB and p-NF-κB decreased in the nucleus with 100 nM α-Syn treatment. Significant differences in expression of NF-κB were observed between control vs. LPS treatment and α-Syn treatment vs. LPS treatment, as LPS treatment promoted translocation of NF-κB (Fig. 3i, k). In contrast, PPARγ, a transcription factor that promotes an anti-inflammatory phenotype, was higher in the 100 nM monomeric α-Syn treatment condition (Figs. 3h and s6g). Moreover, the complex IKKa/β and p-IKB-α, both involved in activation and phosphorylation of NF-κB, decreased under monomeric α-Syn treatment (Figs. 3h and s6b-f). Together, these observations suggest that α-Syn monomer mediates microglial anti-inflammatory phenotype via ERK, NF-κB, and PPARγ.

### Injection of monomeric α-Syn into SNCA-KO mice regulates microglia toward an anti-inflammatory phenotype

**Fig. 4:**
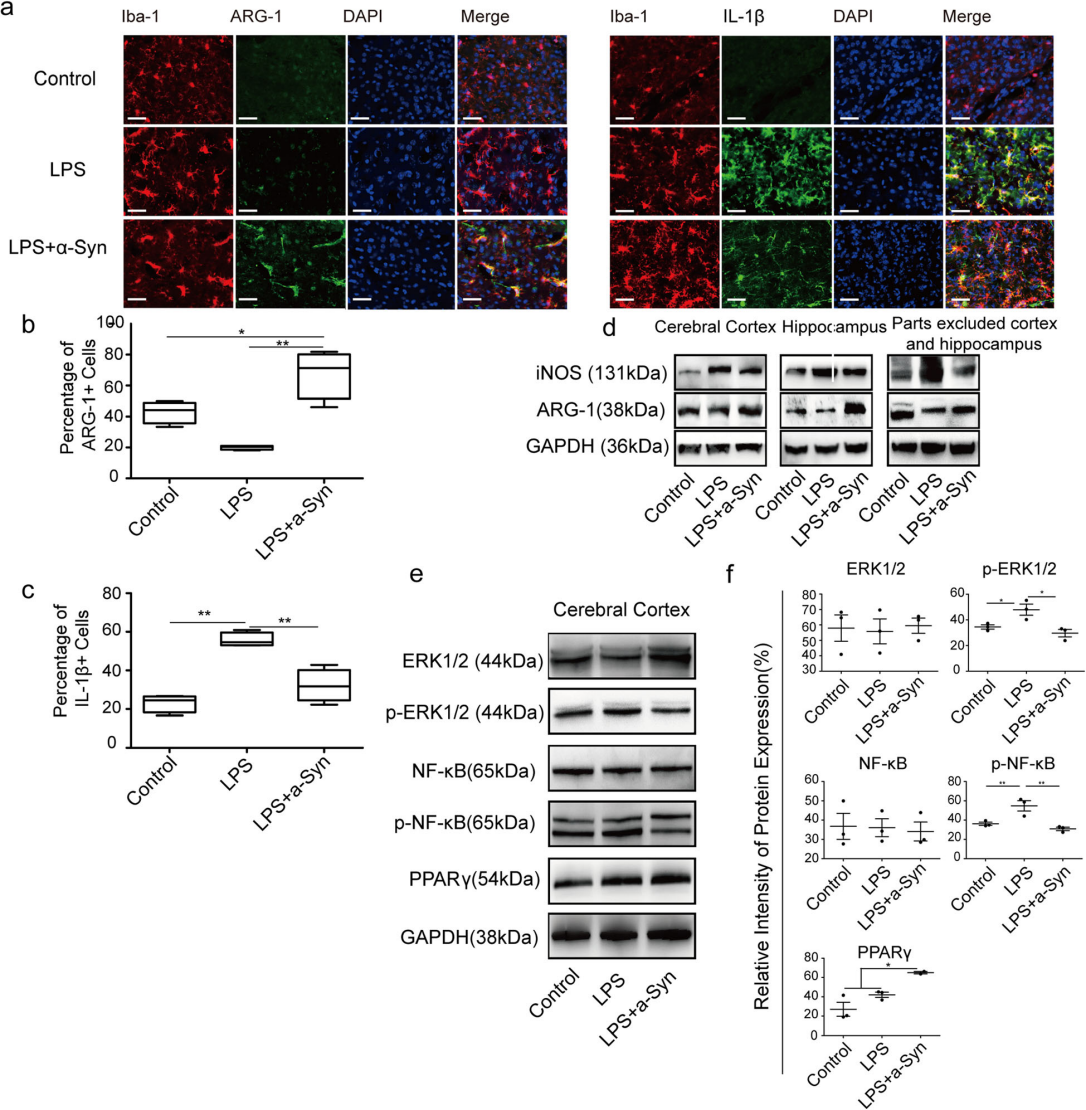
α-Syn injection regulates microglia toward an anti-inflammatory phenotype in vivo. **a** α-Syn increases ARG-1 (green) expression and decreases IL-1β (green) in Iba-1 (red) positive microglia in cerebral cortex by IF (scale bar = 10 μm, *n* = 5 mice). **b** Quantification of ARG-1 positive microglia in brain. **c** Quantification of IL-1β positive microglia in brain. **d** α-Syn upregulates ARG-1 and downregulates iNOS expression in the cerebral cortex compared with that in hippocampus and the remainder of the brain excluding cerebral cortex and hippocampus. (*n* = 3 independent experiments). **e** Detection of ERK-NF-Κb/PPARγ pathway related molecules in the cerebral cortex was consistent with their trends in primary microglia (*n* = 5). **f** Densitometric analyses of relative intensity of ERK, p-ERK, NF-κB, p-NF-κB, and PPARγ expression normalized to GAPDH. One-way ANOVA with Newman Keuls multiple comparison test. Bar graphs show mean + s.e.m.*p < 0.05; **p < 0.01

We next sought to determine whether application of monomeric α-Syn could modulate microglial inflammatory function in vivo in an animal model. To accomplish this goal, without being confounded by endogenously expressed α-Syn, we examined microglial expression of ARG-1 and IL-1β following LPS injection (a condition expected to promote microglial inflammatory function) in mice lacking endogenous α-Syn. This condition resulted in very low levels of cells positive for both Iba-1 and ARG-1 (19.74%), and high levels of Iba-1^+^/ IL-1β^+^ cells (55.71%). However, when both LPS and monomeric α-Syn were injected (a combination that has previously been shown to result in the rapid infiltration of α-syn from the blood to the brain via disruption of the blood-brain barrier [41]), monomeric α-syn could be observed in the brains of α-Syn KO animals (Fig. s1e). When microglia of α-Syn monomer-injected animals were observed, the percentage of Iba-1^+^/ARG-1^+^ cells dramatically increased (67.66%), while the percentage of Iba-1^+^/ IL-1β^+^ cells decreased (32.11%), suggesting that exogenously applied monomeric α-Syn was able to shift the microglia toward an anti-inflammatory phenotype in vivo (Fig. 4a-c). The levels of microglia phenotype-related markers including iNOS and ARG-1 in different encephalic regions were also examined. While animals injected with only LPS exhibited high levels of iNOS, addition of LPS + α-Syn monomer together both diminished iNOS and increased ARG-1 (Fig. 4d).

The molecular signaling pathways shown to be involved in α-syn-mediated inflammatory modulation in vitro were measured in mouse brain tissue, and showed a trend consistent with that found in primary microglia (Fig. 4e). The relative intensity of p-ERK1/2, p-NF-κB, and PPARγ expressions were statistically different among groups (Fig. 4f).

### α-Syn promotes elimination of microglial inflammation and protects against neuronal loss induced by MPTP

**Fig. 5:**
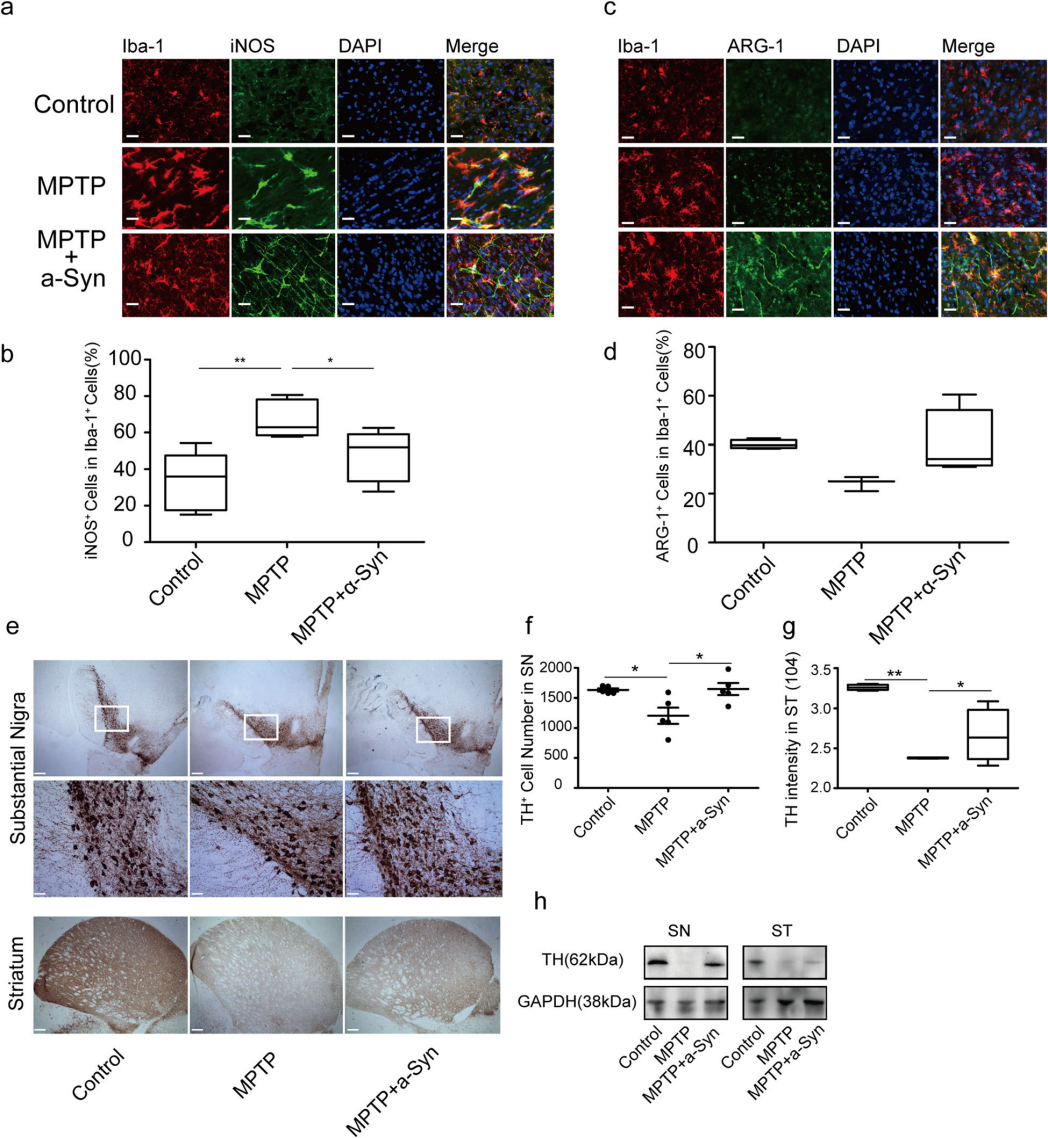
Co-injection of monomeric α-Syn rescues TH^+^ neuron survival in the MPTP model which may be related to the elimination of microglial neuroinflammation induced by MPTP. **a** Immunofluorescent staining for iNOS (green) and Iba-1 (red) in microglia, and nuclei (blue) (scale bar = 10 μm, *n* = 5 mice). **b** Percentage of iNOS^+^ cells in Iba-1^+^ microglia. **c** Immunofluorescence staining for ARG-1 (green) and Iba-1 (red) in microglia, and DAPI (blue) (scale bar = 10 μm, *n* = 5 mice). **d** Percentage of ARG-1^+^ cells in Iba-1^+^ microglia. **e** Immunohistochemical staining for tyrosine hydroxylase (TH) in the SN and striatum. Higher magnification of the areas highlighted by the boxes in the top row are shown in the second row. (Upper scale bar = 1 mm, lower scale bar = 100 μm, *n* = 5 mice). **f** Quantification of TH^+^ cells in SN. **g** Quantification of TH level in ST. **h** TH expression level in SN and ST of each group (*n* = 3 independent experiments from 5 mice). One-way ANOVA with Newman Keuls multiple comparison test. Bar graphs show mean + s.e.m.*p < 0.05

The pro-inflammatory, neurotoxic effects of activated microglia are implicated in neuronal cell death in Parkinson’s disease [16, 35, 42, 43]. Therefore, we also investigated whether modulating of microglia toward an anti-inflammatory phenotype by monomeric α-Syn would have neuroprotective effects in an in vivo mouse model of synucleinopathy. We thus chose a model that features both microglial and α-Syn pathology, in order to examine the potential immunomodulatory effects of monomeric α-Syn in a situation where microglia become activated in an environment of pathological α-syn. We therefore turned to the 1-methyl-4-phenyl-1,2,3,6-tetrahydropyridine (MPTP) model [21, 22, 44], in which microglial NADPH oxidase activity plays a critical role [45]. To accomplish this, we compared dbl-PAC-Tg(SNCAA53T);SNCA^−/−^ animals injected with MPTP to those in which MPTP injection was accompanied by 5 mg/kg of monomeric α-Syn. In the midbrain of MPTP-injected mice, the percentage of iNOS^+^ microglia (67.20%) increased robustly compared with that of control (33.25%) [2], while monomeric α-Syn injection attenuated this effect (47.34%) (Fig. 5a, b). In contrast, ARG-1^+^ microglia remained at high numbers in control (40.12%) and monomeric α-Syn-injected mice (39.95%) (Fig. 5c, d). As expected, MPTP dramatically reduced the number of tyrosine hydroxylase (TH)^+^ neurons observed in the SN. Remarkably, α-Syn monomer injection protected TH^+^ neurons from loss in SN (Fig. 5e) [24], though no significant difference was observed in TH^+^ cell number in SN among control, MPTP mice, and MPTP + α-Syn monomer mice (Fig. 5f). Injection of monomeric α-Syn also preserved TH expression in SN and ST (Fig. 5g-i). P-ERK, NF-κB, and p-NF-κB were all significantly inhibited, along with promotion of PPARγ by α-Syn injection (Figs. 5j and s7), demonstrating successful shifting of microglia toward an anti-inflammatory phenotype. Together, these results suggest that manipulation of the balance in pro-inflammatory vs. anti-inflammatory microglia by monomeric α-Syn was beneficial to neuronal survival.

## Discussion

The most important discovery of this study is revealing a novel modulator of physiological microglial functions, i.e., inhibition of their pro-inflammatory phenotype by monomeric α-Syn. As a gene important to Parkinson’s disease and related LB pathology, pathological roles of α-Syn have been clearly implicated [46, 47], including in the provocation of pro-inflammatory phenotypes of microglia by aggregated/oligomeric forms. In contrast, maintenance of physiological, anti-inflammatory, and beneficial effect phenotypes of microglia is a novel function of α-Syn, despite its relatively high protein levels and widespread CNS expression. Its suggested functions include effects in promoting ATP synthase efficiency [48], and regulation of vesicular release at the synapse [49], neuronal excitability [50], and modulation of lipid metabolism [51], through its lipid-binding properties. Further, the protein is capable of assuming a variety of conformations, including monomeric, tetrameric in some studies but not others [26, 52], oligomeric, and aggregated forms [12], though the relative contributions to the total concentrations, as well as potential distinct functions, are not fully characterized. It is known that oligomeric or aggregated α-Syn has potent activity in activating microglia [16, 27]. However, the effects observed in this study were most likely provoked by the monomer (Fig. s1a). This is, to our knowledge, the first study demonstrating an immunomodulatory function of monomeric α-Syn in directing microglial polarization and or maintaining microglia at physiological state.

In this study, we used concentrations of monomer (100–250 nM) similar to the concentrations of oligomer that have previously been shown to provoke inflammation in microglia [16], but the relevance of this concentration is debatable, and somewhat challenging to assess. While the concentration used is higher than most studies report in cerebrospinal fluid or interstitial fluid or in plasma [53], α-Syn is very abundant in neural tissue, making up as much as 1% of the brain protein [39], and the levels of α-Syn reached locally during neuronal secretion or cell death is unknown. Moreover, given the opposing effects of oligomeric and monomeric α-Syn (see below), it is also possible that the effects in vivo are dependent not only on the absolute concentrations of monomeric and oligomeric forms, but also on their molar ratios.

Microglia play pivotal roles in brain homeostasis. In their so-called “resting” or physiological state, they survey the brain parenchyma, interacting with other cell types, participating in synaptic remodeling, and clearing dead or dying cells by phagocytosis [54]. Multiple studies have confirmed that oligomeric α-Syn activates microglia, resulting in a robust inflammatory response [16, 27, 55]. The current data suggests that pre-exposure to monomeric α-Syn, which is abundant in the extracellular space, e.g., in the cerebrospinal fluid, might suppress activation by oligomers (Fig. 2). This suggests that the earliest stages of oligomer exposure may be counterbalanced by exposure to pre-existing α-Syn monomer (and potentially, other physiological forms), with the effects of aggregated α-Syn needing to overcome the counteracting effects of monomer before pro-inflammatory activity occurs in vivo. Intriguingly, our demonstration of the disparate effects of oligomeric vs. monomeric α-Syn on microglial activation is consistent with a previous finding that monomeric α-Syn enhances microglial phagocytosis behavior, as expected in cells transformed to a protective phenotype [56]. Notably, as discussed earlier, while the pro- and anti-inflammatory functions of microglia are classically categorized as belonging to distinct phenotypes (M1 and M2, respectively), a modern view of microglial polarization is more nuanced, recognizing that the states are overlapping and occur on a spectrum, rather than as a simple binary, and pro- or anti-inflammatory responses are actually intermingled [57, 58], with multiple subtypes of microglia performing more specialized functions within each of the broader categories. Thus, further studies will be needed to fully characterize the active state promoted by physiological α-Syn, as well as the extent of its functions in the brain.

We further examined the mechanism by which monomeric α-Syn promotes an anti-inflammatory phenotype. Previously published results demonstrated that α-Syn interacts with ERK [38]. Our present experiments suggest that α-Syn monomer decreases ERK activation, attenuates activation of NF-κB, and increases PPARγ expression (Fig. 3a, i), with levels of p-ERK decreased in α-syn-treated microglia in vitro and in MPTP-treated mice (Figs. 5 and s7). Because ERK activation has roles in both pro- and anti-inflammatory actions of microglia [59–61], we also examined the effect on cAMP response element-binding protein (CREB), which participates in the anti-inflammatory actions [62]. However, no effect in CREB level or phosphorylation was observed (Fig. s5), suggesting that additional investigations of the actions via ERK are warranted. Further, action of monomeric α-Syn via ERK is particularly interesting in light of the disparate mechanisms of microglial response to α-Syn aggregates. For example, previous studies demonstrated binding of aggregated α-Syn to receptors on the extracellular membrane surface of microglia, while, if the physical interaction with (intracellular) ERK (Fig. 3e) is indeed the mechanism of its action in promoting an anti-inflammatory state, it must first be internalized by the cell. To this end, it should be noted that an early investigation indicates that, in contrast to oligomeric α-Syn, which microglia can take up in a clathrin-dependent mechanism [63], α-Syn monomer can readily enter the microglia, via lipid raft-mediated endocytosis that is clathrin- and caveolae-independent [64], suggesting that these pathways could be plausible therapeutic targets.

In addition to the effects on ERK, treatment of microglia with α-syn attenuated activation of NF-κB and inhibited PPARγ expression (Fig. 3i, j). NF-κB regulates expression of numerous genes and participates in many cellular processes such as production of inflammatory mediators, cell proliferation and survival, differentiation of effector and regulatory T cells, and dendritic-cell maturation [65]. While PPARγ is a member of the nuclear receptor superfamily and confers neuroprotection at several operational levels such as suppression of microglial inflammation, including expression of the microglia inflammatory factors IL-1β, TNF-α, NF-κB, and iNOS [40, 66]. Thus, our findings suggest that monomeric α-Syn not only suppresses the pro-inflammatory phenotype but also actively promotes the protective anti-inflammatory state in microglia. An important remaining question about this mechanism of action, however, is whether similar results would arise from other pathways that activate anti-inflammatory phenotypes, or whether the dampening of the effects of oligomeric α-syn is dependent on monomeric α-syn specifically. This question must be addressed in depth in future studies, particularly given the complex interactions of monomeric α-syn with oligomeric species in the more complex environment of the living brain.

We confirmed the in vitro findings in an in vivo animal model lacking endogenous α-Syn expression, in order to limit confounding by long-term, ongoing exposure to endogenous α-Syn. Intriguingly, addition of monomeric α-Syn prevented conversion of brain microglia to the inflammatory phenotype. While this experiment was effective in demonstrating the principle that α-Syn influences microglial activation in vivo, a number of questions remain when considering the normal physiological state. Indeed, under normal α-Syn-expressing conditions, LPS treatment can initiate progressive loss of dopaminergic neurons, suggesting differing stimuli differentially overcome the influence of physiological α-Syn [67]. Moreover, addition of pathological α-Syn to the model results in an exacerbated, ongoing neuroinflammatory state, further emphasizing the balance of the protective effects of physiological with the deleterious effects of pathogenic forms of α-Syn. Monomeric α-Syn can also cross the BBB when injected intravenously, a process that is enhanced under inflammatory conditions [41]. Given the high endogenous levels of plasma α-Syn, the equilibrium of peripheral α-Syn across the BBB, and the entry of α-Syn into microglia, it is possible that α-Syn that influences microglial behavior may arise from multiple sources, and that control of the entry of peripheral α-Syn into the brain by the BBB could alter it. Thus, microglial phenotype may arise from a complex interaction to which both brain and periphery contribute.

To further demonstrate the effects of monomeric α-Syn on microglial activation, we employed the MPTP model. This protocol produces selective loss of nigrostriatal dopaminergic neurons, driven by neuroinflammatory mechanisms and causing Parkinson’s disease-like symptoms [44, 68–70]. It features robust gliosis [71], and the importance of this feature to the observed pathology is demonstrated by the finding that ablation of the upregulation of iNOS that follows monomeric α-Syn treatment attenuates MPTP neurotoxicity [44]. Further, neuronal death upon exposure to MPTP is greatly exacerbated by microglial production of reactive oxygen species via NADPH oxidase [45]. Our surprising finding that addition of exogenous α-Syn to this system (Fig. 5), which features approximately normal levels of brain α-Syn but highly elevated peripheral α-Syn, strongly attenuates toxicity to dopaminergic cells demonstrates a counterintuitive action of α-Syn in an in vivo model of Parkinson’s disease. Thus, this function may occur primarily due to modulation of the inflammatory effect in the environment of the damaged brain.

Together, these findings suggest that maintenance of the microglial phenotype depends on a balance of the anti-inflammatory, neuroprotective role of the monomer, and the propensity of α-Syn to form neurotoxic oligomers at higher concentrations. Indeed, our own data suggests that the neuroprotective effects may be diminished at the highest concentrations of α-Syn used (Fig 2), in which oligomers would be most likely to form. However, many questions remain, particularly regarding the further interacting influencing factors, some of which may alter α-Syn itself, or the response of microglia to it. Moreover, whether α-Syn derived from the CNS itself, or from the periphery, perform similarly should be considered. Additionally, because inflammation affects the permeability of the BBB to many molecules, including α-Syn itself, the dynamic interaction of microglia with peripheral α-Syn entering the brain might alter responses and the balance between monomer and oligomer (aggregates) as well.

## Conclusions

This work demonstrates a novel physiological function of monomeric α-Syn, largely via promoting microglial anti-inflammatory phenotype, a simplified M2 polarization; these findings increase understanding of the physiological activities of this protein, which has primarily been examined for its pathophysiological roles. Better understanding of the functions of this abundant protein may lead to improved targeting of treatments in the diseases to which it contributes.

## Supplementary information

**Additional file 1: **Figure s1 Identification of α-synuclein (α-Syn)monomer, oligomer and primary microglia purity. **a** α-Syn monomer and oligomer are identified by western blot. Besides monomer, with a molecular weight around 15kDa, oligomer includes dipolymer, tripolymer, tetramer, pentamer with molecular weight ranging from 30kDa to 75kDa. **b** Representative images of immunofluorescence for microglia (Iba-1, red) or astrocyte (GFAP, red), respectively. Nuclei are counterstained with DAPI. (Scale bar=100μm, *n*=5, each containing 10-15 fields). **c** Iba-1^+^ and GFAP^+^ cells are quantified by counting at 10-15 randomly selected fields under each condition (*** *p<0.001*). **d** Forms of α-Syn incubated in media for 6h, 12h and 24h. **e** Forms of injected exogenous α-Syn in both WT and SNCA-KO mice. **f and g** Comparison of anti-inflammatory effect of endogenous α-Syn between WT mice and SNCA-KO mice. (Scale bar=10μm, n=3, each containing 10-15 fields). Figure s2 α-Syn induces microglia towards a pro-inflammatory phenotype. **a** α-Syn induces iNOS expression post 24h treatment in primary microglia (n=3). **b** Densitometric analysis of relative intensity of iNOS expression. **c** Densitometric analysis of relative intensity of ARG-1 expression. **d** Nitrate concentration in supernatant at 24h under different concentrations of α-Syn. **e-h** mRNA levels of ARG-1, CD206, iNOS and CD16/32 in BV2 microglia with 6h treatment at different concentrations. **i-l** mRNA levels of ARG-1, CD206, iNOS and CD16/32 in BV2 microglia with 12h treatment at different concentrations. **m-p** mRNA of ARG-1, CD206, iNOS and CD16/32 in BV2 post treatment by physiological concentration of α-Syn for 24h. **q** Effect of a series concentrations of α-Syn monomer ranging from 0.05nM to 100nM on expression of iNOS and ARG-1 in BV2 microglia. One-way ANOVA with Newman Keuls Multiple Comparison Test. * *p*<0.05, ** *p*<0.01*** *p*<0.001. Bar graphs show mean + s.e.m. Fig. s3. α-Syn oligomer exerts pro-inflammatory effects on microglia in a dose-dependent manner. **a** Oligomeric α-Syn induces iNOS, but not ARG-1 expression in primary microglia in a dose-dependent manner. **b** Densitometric analyses of relative intensities of iNOS and ARG-1 expressions (n=3 independent experiments). c mRNA levels of iNOS and ARG-1 in primary microglia under treatment with oligomer at 5pg/ml and 400pg/ml in BV2 cells (n=3). **d** Immunofluorescent staining of iNOS (red) and ARG-1 (green) in BV2 cells under different treatments (Scale bar=20μm). **e** Quantification of the percentage of iNOS and ARG-1 positive BV2 cells (n=5 replications each containing 15-20 fields). One way ANOVA with Newman Keuls Multiple Comparison Test. Bar graphs show mean + s.e.m. * *p*<0.05, ** *p*<0.01, *** *p*<0.001. Figure s4 Neither α-Syn monomer nor oligomer activated ERK. **a** Primary microglia are treated with physiological concentration of α-Syn or higher concentration (400pg/ml) of oligomer for 12h. **b** Co-localization of α-Syn (green) and ERK (red) in microglia of mouse brain (Scale bar=10μm) Figure s5 Monomeric α-Syn does not alter levels of M2 phenotype-related JAK3-STAT6 pathway, CREB or PPARα. Western blots were performed to determine whether monomeric α-syn altered several pathways, but no major differences were apparent. Figure s6 Quantification of microglia state related molecule expressions by densitometric analysis. **a-g** Relative intensity of ERK, NF-κB, p- NF-κB, IKKα/β, IKBα,p- IKBα,PPARγ post treatment by α-Syn for 12h. One-way ANOVA with Newman Keuls Multiple Comparison Test. * *p*<0.05, ** *p*<0.01*** *p*<0.001. Bar graphs show mean + s.e.m. Figure s7 Co-injection of monomeric α-Syn attenuates microglial inflammation induced by MPTP. Quantitative analyses of protein expression intensities normalized to GAPDH. One-Way ANOVA with Newman Keuls Multiple Comparison Test. Bar graphs show mean + s.e.m.* *p*<0.05

## Data Availability

The data that support the findings of this study are available from the corresponding author upon reasonable request.

## Abbreviations

ARG-1: Arginase 1
CREB: cAMP response element-binding protein
ELISA: Enzyme-linked immunosorbent assay
ERK: Extracellular signal-regulated kinase
FBS: Fetal bovine serum
GFAP: Glial fibrillary acidic protein
IFN-γ: Interferon γ
IL-1β: Interleukin 1 β
IL-18: Interleukin 18
iNOS: Inducible nitric oxide synthase
IP: Immunoprecipitation
i.p.: Intraparetoneal
IV: Intravenous
LB: Lewy body
LPS: Lipopolysaccharide
MPTP: 1-Methyl-4-phenyl-1,2,3,6-tetrahydropyridine
NF-κB: Nuclear factor kappa B
