# Supplementary material for: Immunoregulation of microglial polarization: an unrecognized physiological function of α-synuclein
Source: J Neuroinflammation. 2020 Sep 17;17:272. doi: 10.1186/s12974-020-01940-z (PMC7500012; doi:10.1186/s12974-020-01940-z)
Supplement: Supplementary file 1 — Additional file 1: Figure s1 Identification of α-synuclein (α-Syn)monomer, oligomer and primary microglia purity. a α-Syn monomer and oligomer are identified by western blot. Besides monomer, with a molecular weight around 15kDa, oligomer includes dipolymer, tripolymer, tetramer, pentamer with molecular weight ranging from 30kDa to 75kDa. b Representative images of immunofluorescence for microglia (Iba-1, red) or astrocyte (GFAP, red), respectively. Nuclei are counterstained with DAPI. (Scale bar=100μm, n=5, each containing 10-15 fields). c Iba-1+ and GFAP+ cells are quantified by counting at 10-15 randomly selected fields under each condition (*** p<0.001). d Forms of α-Syn incubated in media for 6h, 12h and 24h. e Forms of injected exogenous α-Syn in both WT and SNCA-KO mice. f and g Comparison of anti-inflammatory effect of endogenous α-Syn between WT mice and SNCA-KO mice. (Scale bar=10μm, n=3, each containing 10-15 fields). Figure s2 α-Syn induces microglia towards a pro-inflammatory phenotype. a α-Syn induces iNOS expression post 24h treatment in primary microglia (n=3). b Densitometric analysis of relative intensity of iNOS expression. c Densitometric analysis of relative intensity of ARG-1 expression. d Nitrate concentration in supernatant at 24h under different concentrations of α-Syn. e-h mRNA levels of ARG-1, CD206, iNOS and CD16/32 in BV2 microglia with 6h treatment at different concentrations. i-l mRNA levels of ARG-1, CD206, iNOS and CD16/32 in BV2 microglia with 12h treatment at different concentrations. m-p mRNA of ARG-1, CD206, iNOS and CD16/32 in BV2 post treatment by physiological concentration of α-Syn for 24h. q Effect of a series concentrations of α-Syn monomer ranging from 0.05nM to 100nM on expression of iNOS and ARG-1 in BV2 microglia. One-way ANOVA with Newman Keuls Multiple Comparison Test. * p<0.05, ** p<0.01*** p<0.001. Bar graphs show mean + s.e.m. Fig. s3. α-Syn oligomer exerts pro-inflammatory effects on microglia in a dose-dependent [file 12974_2020_1940_MOESM1_ESM.docx]

**Supplementary Materials**


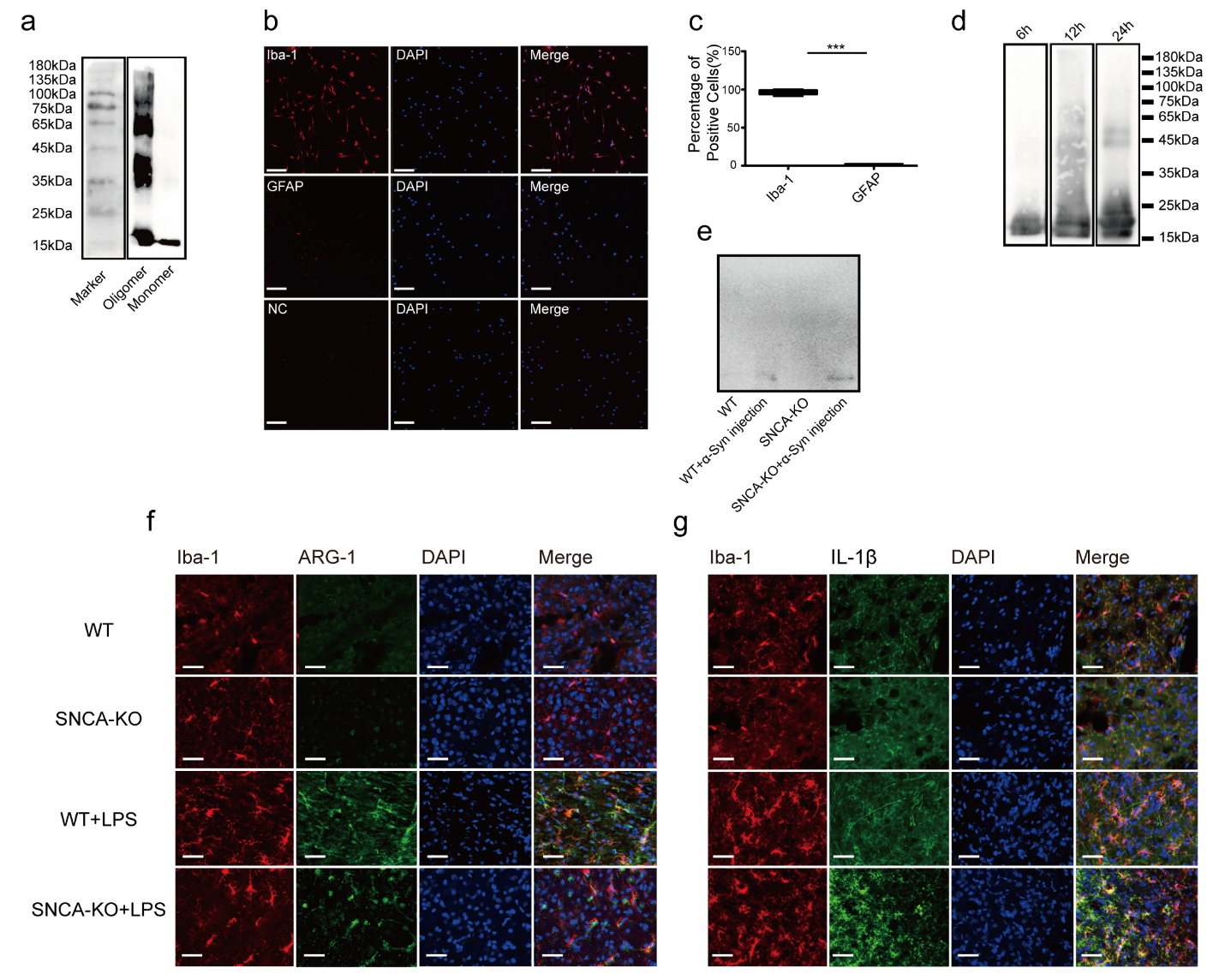


Fig. s1 Identification of α-synuclein （α-Syn）monomer, oligomer and primary microglia purity. **a** α-Syn monomer and oligomer are identified by western blot. Besides monomer, with a molecular weight around 15kDa, oligomer includes dipolymer, tripolymer, tetramer, pentamer with molecular weight ranging from 30kDa to 75kDa. **b** Representative images of immunofluorescence for microglia (Iba-1, red) or astrocyte (GFAP, red), respectively. Nuclei are counterstained with DAPI. (Scale bar=100μm, n=5, each containing 10-15 fields). **c** Iba-1^+^ and GFAP^+^ cells are quantified by counting at 10-15 randomly selected fields under each condition (*** *p<0.001*). **d** Forms of α-Syn incubated in media for 6h, 12h and 24h. **e** Forms of injected exogenous α-Syn in both WT and SNCA-KO mice. **f and g** Comparison of anti-inflammatory effect of endogenous α-Syn between WT mice and SNCA-KO mice. (Scale bar=10μm, n=3, each containing 10-15 fields).


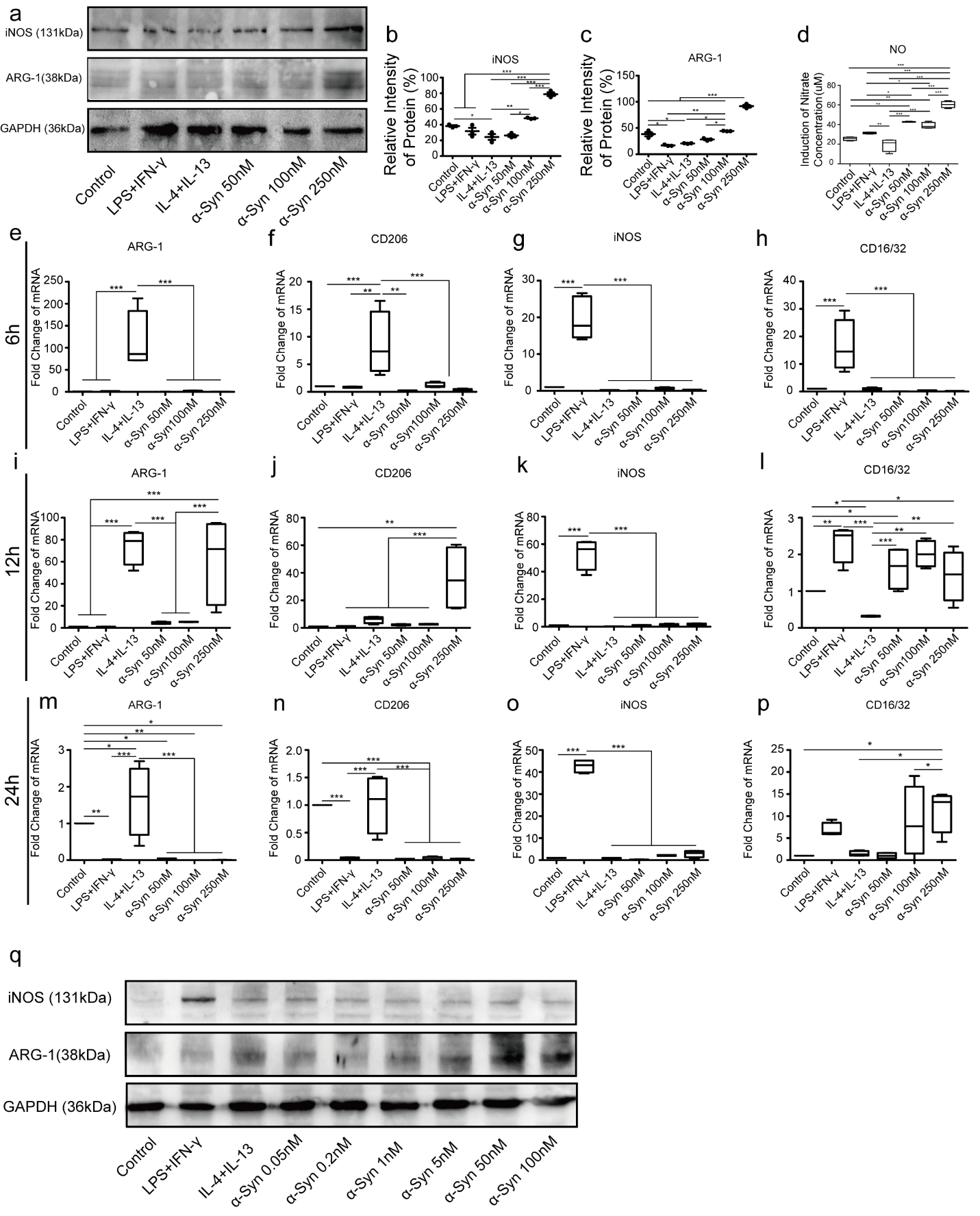


Fig. s2 α-Syn induces microglia towards a pro-inflammatory phenotype. **a** α-Syn induces iNOS expression post 24h treatment in primary microglia (n=3). **b** Densitometric analysis of relative intensity of iNOS expression. **c** Densitometric analysis of relative intensity of ARG-1 expression. **d** Nitrate concentration in supernatant at 24h under different concentrations of α-Syn. **e-h** mRNA levels of ARG-1, CD206, iNOS and CD16/32 in BV2 microglia with 6h treatment at different concentrations. **i-l** mRNA levels of ARG-1, CD206, iNOS and CD16/32 in BV2 microglia with 12h treatment at different concentrations. **m-p** mRNA of ARG-1, CD206, iNOS and CD16/32 in BV2 post treatment by physiological concentration of α-Syn for 24h. **q** Effect of a series concentrations of α-Syn monomer ranging from 0.05nM to 100nM on expression of iNOS and ARG-1 in BV2 microglia. One-way ANOVA with Newman Keuls Multiple Comparison Test. * *p*<0.05, ** *p*<0.01*** *p*<0.001. Bar graphs show mean $+$ s.e.m.


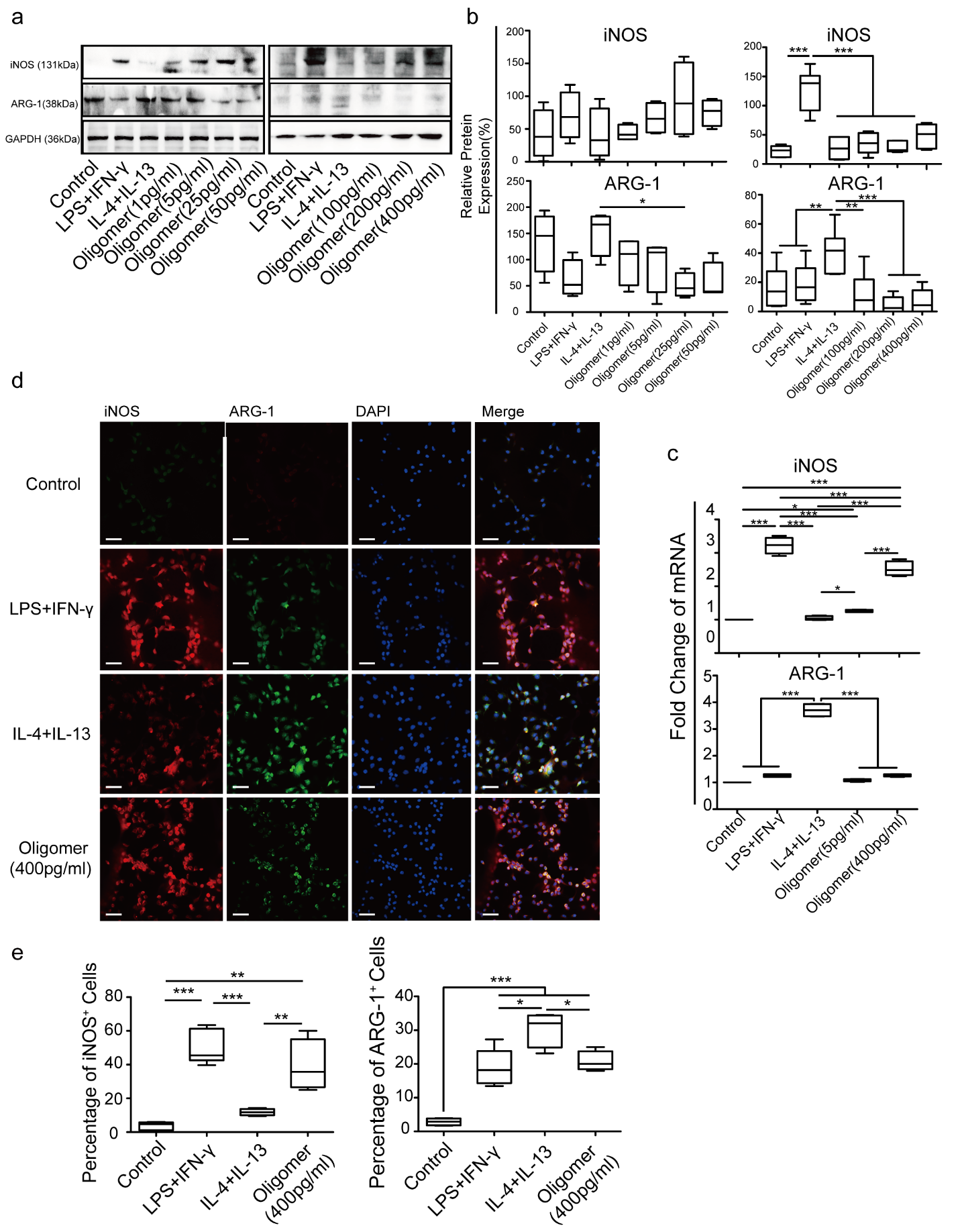


Fig. s3. α-Syn oligomer exerts pro-inflammatory effects on microglia in a dose-dependent manner. **a** Oligomeric α-Syn induces iNOS, but not ARG-1 expression in primary microglia in a dose-dependent manner. **b** Densitometric analyses of relative intensities of iNOS and ARG-1 expressions (n=3 independent experiments). c mRNA levels of iNOS and ARG-1 in primary microglia under treatment with oligomer at 5pg/ml and 400pg/ml in BV2 cells (n=3). **d** Immunofluorescent staining of iNOS (red) and ARG-1 (green) in BV2 cells under different treatments (Scale bar=20μm). **e** Quantification of the percentage of iNOS and ARG-1 positive BV2 cells (n=5 replications each containing 15-20 fields). One way ANOVA with Newman Keuls Multiple Comparison Test. Bar graphs show mean $+$ s.e.m. * *p*<0.05, ** *p*<0.01, *** *p*<0.001


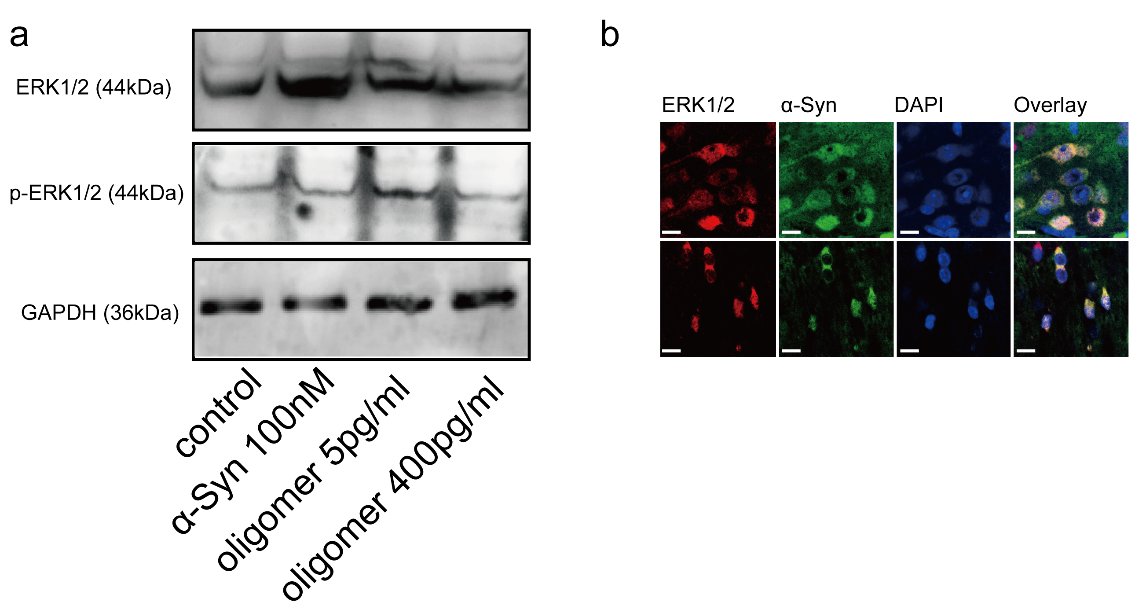


Fig. s4 Neither α-Syn monomer nor oligomer activated ERK. **a** Primary microglia are treated with physiological concentration of α-Syn or higher concentration (400pg/ml) of oligomer for 12h. **b** Co-localization of α-Syn (green) and ERK (red) in microglia of mouse brain (Scale bar=10μm).


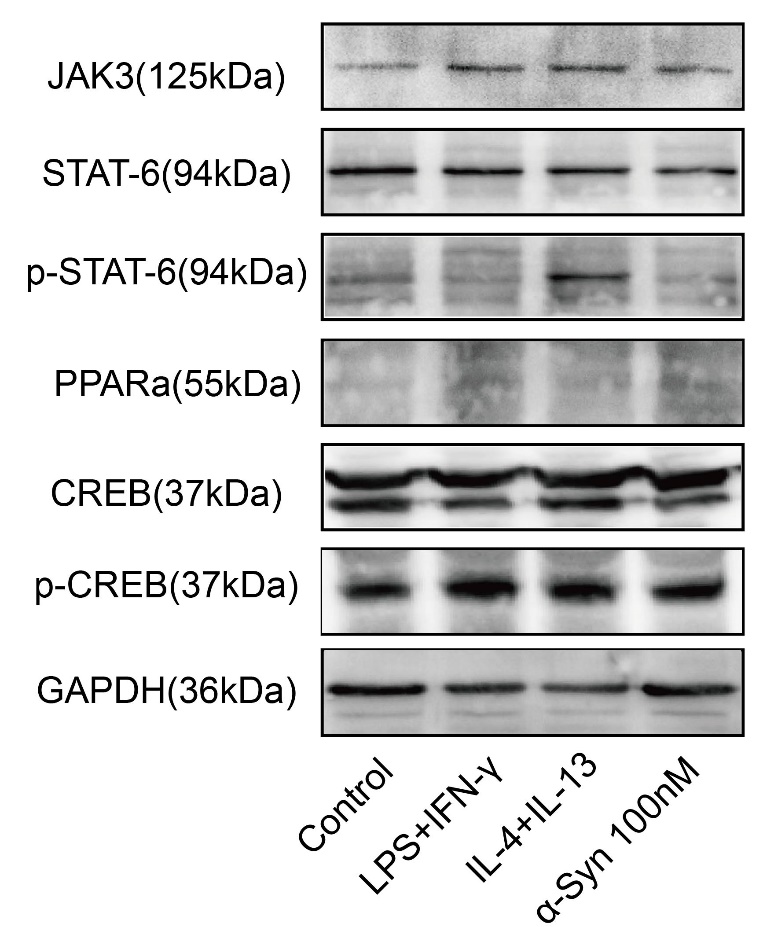


Fig. s5 Monomeric α-Syn does not alter levels of M2 phenotype-related JAK3-STAT6 pathway, CREB or PPARα. Western blots were performed to determine whether monomeric α-syn altered several pathways, but no major differences were apparent.


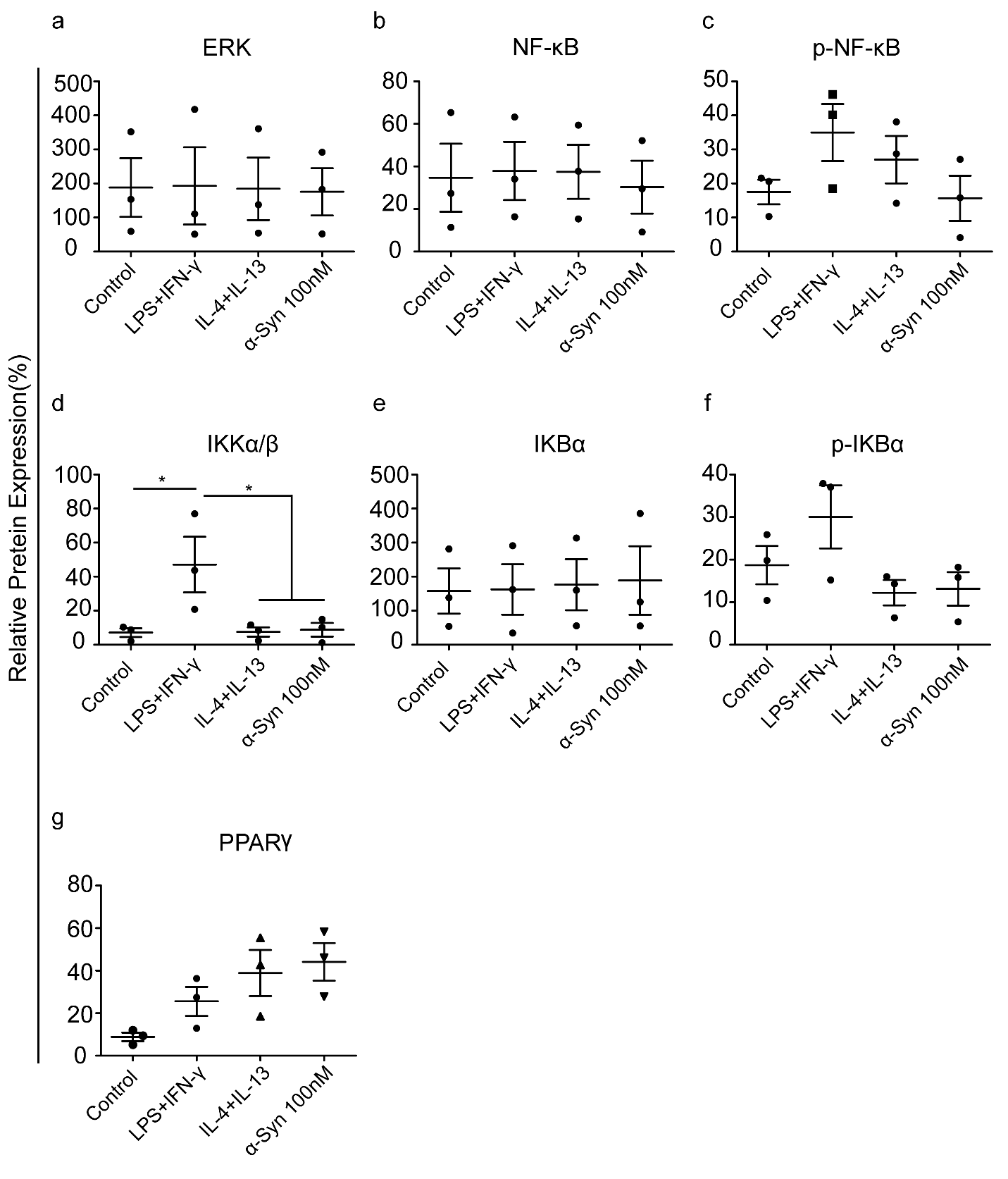


Fig. s6 Quantification of microglia state related molecule expressions by densitometric analysis. **a-g** Relative intensity of ERK, NF-κB, p- NF-κB, IKKα/β, IKBα,p- IKBα,PPARγ post treatment by α-Syn for 12h. One-way ANOVA with Newman Keuls Multiple Comparison Test. * p<0.05, ** p<0.01*** p<0.001. Bar graphs show mean $+$ s.e.m

**
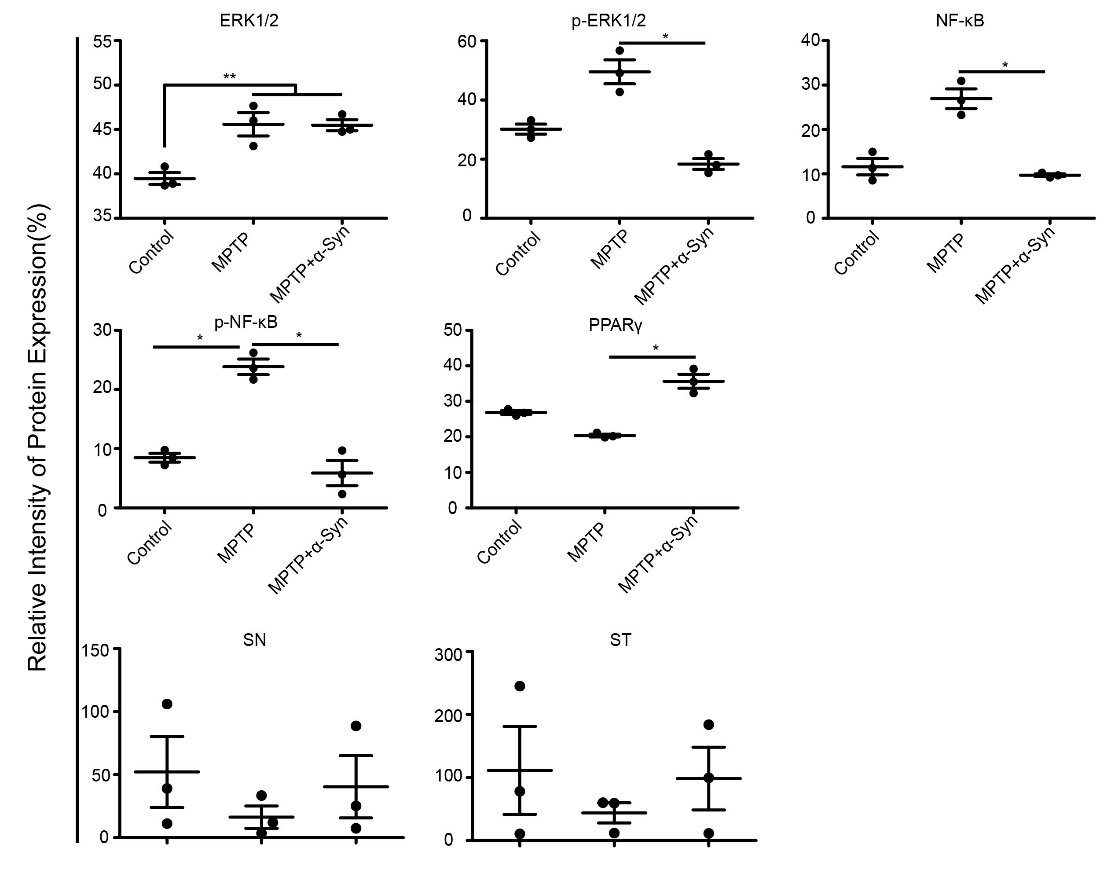
**

Fig. s7 Co-injection of monomeric α-Syn attenuates microglial inflammation induced by MPTP. Quantitative analyses of protein expression intensities normalized to GAPDH. One-Way ANOVA with Newman Keuls Multiple Comparison Test. Bar graphs show mean $+$ s.e.m.* *p*<0.05
